# Supplementary material for: Resolving the full spectrum of human genome variation using Linked-Reads
Source: Genome Res. 2019 Apr;29(4):635–45. doi: 10.1101/gr.234443.118 (PMC6442396; doi:10.1101/gr.234443.118)
Supplement: Supplemental Material [file supp_gr.234443.118_Supplemental_longranger-2.2.zip › longranger-2.2.tar.bz2!/lr2.2-public/lib/stan/cmdstan/stan/doc/api/html/math_2functions_2lmgamma_8hpp_source.html]

Stan: src/stan/math/functions/lmgamma.hpp Source File


|  |  |
| --- | --- |
|  | Stan  2.5.0  probability, sampling & optimization |


- Main Page
- Related Pages
- Namespaces
- Classes
- Files

- File List
- File Members

All Classes Namespaces Files Functions Variables Typedefs Enumerations Enumerator Friends Macros Pages

- src
- stan
- math
- functions

lmgamma.hpp

Go to the documentation of this file.

1 #ifndef STAN\_\_MATH\_\_FUNCTIONS\_\_LMGAMMA\_HPP

2 #define STAN\_\_MATH\_\_FUNCTIONS\_\_LMGAMMA\_HPP

3

4 #include <boost/math/tools/promotion.hpp>

5 #include <stan/math/constants.hpp>

6 #include <boost/math/special\_functions/gamma.hpp>

7

8 namespace stan {

9

10  namespace math {

11

55  template <typename T>

56  inline typename boost::math::tools::promote\_args<T>::type

57  lmgamma(const int k, T x) {

58  using boost::math::lgamma;

59  typename boost::math::tools::promote\_args<T>::type result

60  = k \* (k - 1) \* LOG\_PI\_OVER\_FOUR;

61

62  for (int j = 1; j <= k; ++j)

63  result += lgamma(x + (1.0 - j) / 2.0);

64  return result;

65  }

66

67  }

68 }

69 #endif

stan::agrad::lgamma

fvar< T > lgamma(const fvar< T > &x)

**Definition:** lgamma.hpp:15

stan::math::lmgamma

boost::math::tools::promote\_args< T >::type lmgamma(const int k, T x)

Return the natural logarithm of the multivariate gamma function with the speciifed dimensions and arg...

**Definition:** lmgamma.hpp:57

stan::math::LOG\_PI\_OVER\_FOUR

const double LOG\_PI\_OVER\_FOUR

**Definition:** constants.hpp:70

stan::math::lgamma

double lgamma(double x)

**Definition:** lgamma.hpp:31

constants.hpp


---

[ Stan Home Page ]

*© 2011–2014,
Stan Development Team.*
